# Retinal blood oxygen saturation and vascular endothelial growth factor-A in early diabetic retinopathy: A protocol for systematic review and meta-analysis

**DOI:** 10.1097/MD.0000000000020562

**Published:** 2020-06-12

**Authors:** Xianliang Qiu, Xian Wang, Peipei Hong, Min Liu, Qing Wen, Qiu Chen

**Affiliations:** a School of Clinical Medicine, Hospital of Chengdu University of Traditional Chinese Medicine; b Department of Endocrinology, Hospital of Chengdu University of Traditional Chinese Medicine, Chengdu, China.

**Keywords:** blood oxygen saturation, diabetes mellitus, retinal disease, type 2, vascular endothelial growth factor

## Abstract

**Background:**

Diabetic retinopathy (DR) remains the most common microvascular complication of diabetes; both its high prevalence and associated high risk of vision loss lead it to the major global health burden. Despite significant research efforts, there still remains much of the underlying pathology not fully understand. In the past studies, inner retinal blood flow disturbances are widely assessed as a potential biomarker of DR. However, the results have been variable and even contradictory. Improved methods to figure out the metabolic disturbances associated with DR are essential. Some research showed that both vascular endothelial growth factor-A and blood oxygen saturation are higher in DR patients and correlated with disease severity. Therefore, we decided to conduct a systematic review and meta-analysis to find out the connection of them and provide robust evidence on the mechanism underlying this connection to help us better understand the pathophysiology behind the hypoxic retina and find better ways to treat DR.

**Methods:**

This study will be conducted according to Preferred Reporting Items for Systematic Review and Meta-Analysis Protocols. We will do electronic searches from PubMed, EMBASE, Web of Science, Cochrane Library, Wanfang database, CNKI, and VIP database. Two reviewers will screen all the references independently and any disagreement will be solved by the third involvement. All data will be extracted by 2 independent reviewers according to a standardized data extraction sheet. Based on the type of studies, 2 reviewers will independently use different scales to assess the risk of bias. Any disagreements and conflicts will be resolved by discussing it with a third reviewer. We will conduct a random-effects meta-analysis. Individual and pooled odds ratios/relative risks and associated 95% confidence intervals will be calculated as well as between-study heterogeneity. The potential for publication bias will also be evaluated. If possible, we will explore reasons for potential between-study heterogeneity.

**Results:**

This study is a protocol for systematic review and meta-analysis without results. Associated data analysis will be carried out after the protocol.

**Conclusion:**

The protocol aims to guide a meta-analysis whose purpose is investigating the association between retinal blood oxygen saturation and vascular endothelial growth factors in patients with early DR and trying to find out the mechanism that the changing of retinal oxygen saturation in patients with DR.

**INPLASY registration number:**

INPLASY202040161

## 1 Introduction

Diabetic retinopathy (DR) remains the most common microvascular complication of diabetes; both its high prevalence and associated high risk of vision loss in the working age population lead it to the major global health burden.^[1]^ In general, DR can be split into different stages according to whether there are new vessels. In the absence of new vessels, DR is classified as nonproliferative (NPDR) which ranges from mild to severe in severity. In the presence of new vessels, DR will be considered to be proliferative DR (PDR).^[2]^ Without treatment, DR progresses will develop from mild NPDR to moderate and severe NPDR, finally convert to proliferative DR (PDR).^[3]^ Despite significant research efforts, there still remains much of the underlying pathology not fully understand in this disease. In the past studies, inner retinal blood flow disturbances are widely assessed as a potential biomarker of DR. However, the results have been variable and even contradictory.^[4–7]^ Improved methods to figure out the metabolic disturbances associated with DR are essential.

The present results show that DR is a direct consequence of retinal oxygen metabolism disorder initiated by hyperglycemia.^[8]^ Since the development of fluorescein angiography, hypoxia has been known to occur in DR which has demonstrated that the widespread obliteration of retinal capillaries must leave parts of the inner retina with a much reduced blood supply and caused the ischemic proliferative in retinal, in the later stages of the condition.^[9]^ With abundant experimental and clinical evidence, we can figure out that the mentioned proliferative response is driven by vascular endothelial growth factor (VEGF). VEGF is a potent, diffusible, endothelial-specific mitogen. Under the condition of hypoxia, VEGF will be released and upon binding to the VEGF receptor 2 (VEGFR-2), expressed by the vascular endothelium, elicits angiogenesis and vascular hyperpermeability.^[10]^

The VEGF family includes VEGF-A, VEGF-B, VEGF-C, VEGF-D, VEGF-E, and placenta growth factors 1 and 2. As the primary member that VEGF-A has been considered for a major contributor to the development of DR by: promoting proliferation, differentiation, division, and migration of the vascular endothelial cells; inducing tube formation; and maintaining and stabilizing the newly formed blood vessels.^[11]^ Besides, upregulation of VEGF-A was detected in plasma and vitreous in PDR patients, and showed an increased pattern as the severity of DR increased.^[12]^ However, all above is a (relatively) late stage in DR, and such pathological changes that cannot shed light upon the early causes which we will investigate in followed study.

Blood oxygen saturation is one of the practical factors of the microcirculation that can reflect retinal metabolic activity. Some research^[8,13–15]^ has demonstrated that retinal vessel oxygen saturation is higher in all categories of DR compared with healthy subjects, which may conflict with the traditional concept of DR as an ischemic disease. However, the observation may be explained by followed items^[16]^: compensatory increased oxygen supply in the retina, poor oxygen distribution to the tissue, or lower oxygen consumption in the retinal tissue. Among others, it has been speculated that patients may have a poor oxygen distribution to the ischemic retinal tissue caused by thickening of the capillary walls, capillary nonperfusion, higher affinity for oxygen in glycosylated hemoglobin, and arteriovenous shunting.^[16,17]^ In addition, the viewpoint^[13,17]^ that the retinal vessel oxygen saturation increased with severity of retinopathy has been verified.

In conclusion, high retinal oxygen demand tends to the particular vulnerability of the retina to vascular disease in DR.^[18]^ Pathologic transformations of the retinal vasculature, from permeability to remodeling to neovascularization, are associated with increased expression of VEGF-A.^[10]^ Both VEGF-A and blood oxygen saturation are higher in DR patients and correlated with disease severity. Therefore, we decided to conduct a systematic review and meta-analysis to find out the connection of them and provide robust evidence on the mechanism underlying this connection. The result of this study may help us to better understand the pathophysiology behind the hypoxic retina and find better ways to treat DR.

## 2 Objectives

The aim of this study was to investigate the association between retinal blood oxygen saturation and vascular endothelial growth factors in patients with DR and trying to find out the mechanism that the changing of retinal oxygen saturation in patients with DR in early stage.

## 3 Methods

The present protocol has been registered in the INPLASY (registration number INPLASY202040161; DOI:10.37766/inplasy2020.4.0161) which could be available on https://inplasy.com/inplasy-2020–4–0036/. The article will be reported in accordance with the Preferred Reporting Items for Systematic Review and Meta-Analysis Protocols^[19]^ statement.

### 3.1 Type of studies

#### 3.1.1 Inclusion criteria:

Type of studies cross-sectional, case–control, and both prospective and retrospective cohort-studies will be included if:

1. They investigate the association between retinal blood oxygen saturation and VEGF-A;
2. They report data on retinal blood oxygen saturation and VEGF;
3. They focus on NPDR population.

#### 3.1.2 Exclusion criteria

Studies will be excluded if:

1. They are not written in English or Chinese;
2. They focus on PDR population;
3. They only reported data on retinal blood oxygen saturation or VEGF
4. They are randomized controlled trials (aimed to exclude the potential bias of treatment interventions).

### 3.2 Type of participants

We will include patients aged ≥18 years with type 2 diabetes mellitus and has been diagnosed with NPDR in observational studies (cross-sectional, case–control, and cohort), regardless of sex and ethnicity.

### 3.3 Outcomes

The primary outcome is the association between retinal blood oxygen saturation and VEGF-A. The additional outcomes are the relationship between the retinal blood oxygen saturation and sociodemographic characteristics (eg, age, sex, ethnicity, BMI, life style) or other aqueous humour biomarkers (eg, Ang2, EGF, HGF, IL-8).

### 3.4 Search methods for the identification of studies

#### 3.4.1 Electronic searches

**Figure 1:**
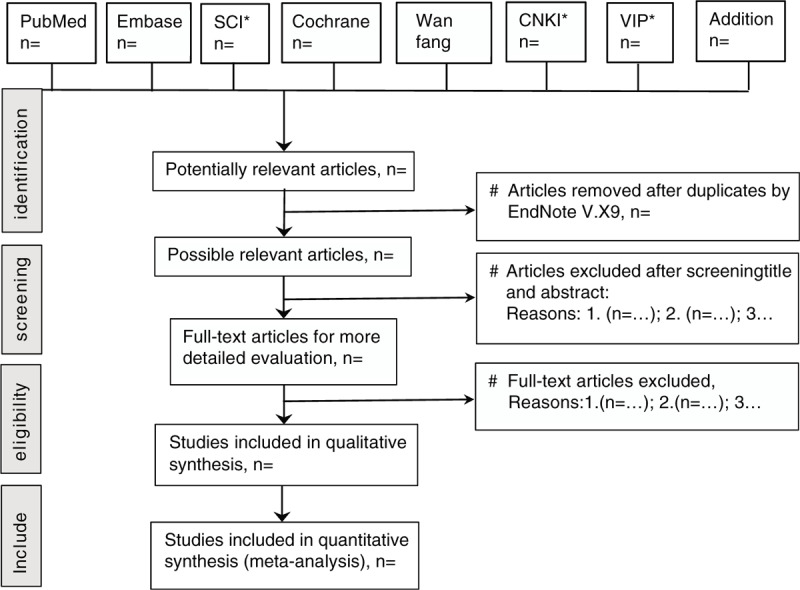
Study selection flow chart.

We will do electronic searches from English databases (PubMed, EMBASE, Web of Science, Cochrane Library) and Chinese databases (Wanfang database, CNKI, and VIP database) from their inception until present, with the Combinations of Medical Subject Headings terms (Table 1): [Retinal blood oxygen saturation] AND [Vascular Endothelial Growth Factor-A OR VEGF-A OR Vasculotropin OR VEGF OR Vascular Endothelial Growth Factor OR Vascular Permeability Factor OR Permeability Factor, Vascular OR Glioma-Derived Vascular Endothelial Cell Growth Factor OR Glioma Derived Vascular Endothelial Cell Growth Factor OR GD-VEGF] AND [Diabetic Retinopathy OR Diabetic Retinopathies OR Retinopathies, Diabetic OR Retinopathy, Diabetic] (Fig. 1).

**Table 1:**
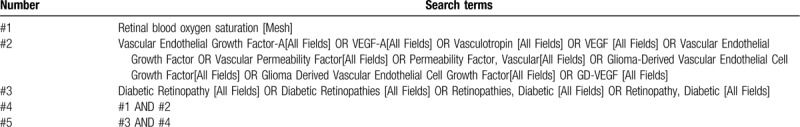
PubMed search strategy.

#### 3.4.2 Reference lists

The reference lists of all the included studies, relevant articles, and previous systematic reviews will be also hand-searched and analyzed for the identification of additional studies.

What's more, we will manually search the database of Chengdu University of Traditional Chinese Medicine Library and our hospital's experts in endocrinology and ophthalmology will be consulted, too.

### 3.5 Data collection

#### 3.5.1 Selection of studies

At the first stage, all studies generated by the search strategies will be imported into EndNote V.X9 and removed duplicates by this software. At next stage, the titles and abstracts of all the references will be identified to check by our 2 reviewers (XQ and PH) if they meet the selection criteria. Then all studies potentially qualified for inclusion will be in addition to the preliminary list and the same 2 reviewers will retrieve their full-text articles. All full text will be evaluated to verify whether they meet the inclusion criteria. In addition, if there goes some disagreement in our 2 reviewers, the final decision will be made by consensus with the involvement of Wen or Liu.

#### 3.5.2 Data extraction and management

Using a standardized data extraction sheet created in Microsoft Office Excel V.2010. To avoid the risk of overlapping studies for each study, we will extract the names of the authors/databases/studies (data source) and merge them using EndNote V.X9 to facilitate checking duplicates. After that, Qiu and Hong will independently extract data from the included studies. Any disagreement will be discussed with a third member of the review team (QW or ML), and decisions will be documented. In the case of missing information concerning the outcomes of interest, we will directly contact study authors up to 3 times to obtain additional information.

We will extract all the data from selected studies: country, year, sample size and type, study design, percentage of males, age, BMI, ethnicity, period of DR, life style (smoking/alcohol), list of confounders included in design and analysis, crude numbers and measure of association (odds ratios/relative risks [ORs/RRs]) and 95% confidence intervals (CIs), and data source.

#### 3.5.3 Risk of bias (quality) assessment in included studies

Two reviewers (XQ and PH) will independently use different scales to assess the risk of bias based on the type of studies: cross-sectional studies will be assessed by the Agency for Healthcare Research and Quality recommended criteria (Table 2).^[20]^ The criteria include 11 items, answered by “yes,” “no,” “unclear”; case–control and cohort studies will use the Newcastle-Ottawa Scale^[21]^ which assess the quality of studies with 8 questions in 3 broad categories: patient selection; comparability of study groups; assessment of the outcome. The evaluation will use the semiquantitative principle of the star system and the highest score is 9 stars. Stars of 0 to 4 mean low quality and 5 to 9 mean high quality. Any disagreements will be solved by discussion or with arbitrament by the third reviewers QW or ML.

**Table 2:**
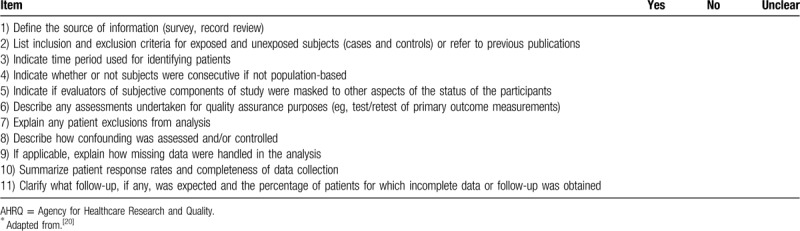
AHRQ recommended cross-sectional quality criteria^∗^.

## 4 Data analyses

### 4.1 Main analyses

We will calculate pooled ORs/RRs and 95% CIs. We will assess heterogeneity with the *χ*^2^ goodness of fit and *I*^2^ statistics. Concerning *I*^2^, we will consider Cochrane recommendations. We will think statistically significant a *P* value < .05 (presence of heterogeneity). In the case of the presence of heterogeneity, we will perform sensitivity analyses and retrogression when possible. When *I*^2^ <50% and *P* > .1, the fixed model will be conducted. Otherwise, the random-effects model is about to be employed for meta-analysis. Publication bias will be determined by a funnel plot and we will try to interpret funnel plot asymmetry by Egger linear regression test if funnel plots are asymmetric.

### 4.2 Additional analyses

If there is a sufficient number of studies for each DR, we will investigate potential sources of heterogeneity using metaregression models, and we will perform subgroup analyses by sociodemographic characteristics (eg, age, sex, ethnicity, BMI, life style) and other aqueous humour biomarkers (eg, Ang2, EGF, HGF, IL-8) and by study design (cross-sectional, case–control, and cohort study).

### 4.3 Software

All analyses will be conducted in Stata V. 14.

### 4.4 Patient and public involvement

Patients and/or public were not involved.

### 4.5 Ethics and dissemination

No ethical approval is required to perform this study. We will publish results in a peer-reviewed scientific journal and data set will be made freely available.

## 5 Strengths and limitations of this study

To our knowledge, this will be the first systematic review and meta-analysis investigating the relationship between retinal blood oxygen saturation and vascular endothelial growth factor-A in early DR. The implications of our future result might help us to understand the pathophysiology behind the hypoxic retina and further study of the basic mechanisms controlling intraocular angiogenesis to provide better ways for treating DR.

Limitations mainly included: the number of studies meeting our screening criteria could be limited; also, it could be difficult to distinguish the correlation results which could be co-variation or causation.

## Author contributions

**Conceptualization:** Xianliang Qiu, Xian Wang, Qiu Chen.

**Data curation:** Xianliang Qiu, Peipei Hong, Qing Wen, Min Liu.

**Investigation:** Xianliang Qiu, Peipei Hong.

**Methodology:** Xianliang Qiu, Xian Wang.

**Software:** Xianliang Qiu.

**Supervision:** Qiu Chen.

**Writing – original draft:** Xianliang Qiu.

**Writing – review:** Qiu Chen, Xian Wang.

QC is the guarantor. All authors read, provided feedback and approved the final manuscript.

## References

[1] Cheung N Mitchell P Wong TY Diabetic retinopathy. Lancet 2010;376:124–36. 20580421 10.1016/S0140-6736(09)62124-3

[2] El Rami H Barham R Sun JK Evidence-based treatment of diabetic retinopathy. Semin Ophthalmol 2017;32:67–74. 27700224 10.1080/08820538.2016.1228397

[3] Wong TY Cheung CM Larsen M Diabetic retinopathy. Nat Rev Dis Primers 2016;2:16012. 27159554 10.1038/nrdp.2016.12

[4] Grunwald JE DuPont J Riva CE Retinal haemodynamics in patients with early diabetes mellitus. Br J Ophthalmol 1996;80:327–31. 8703884 10.1136/bjo.80.4.327 PMC505459

[5] Bursell SE Clermont AC Kinsley BT Retinal blood flow changes in patients with insulin-dependent diabetes mellitus and no diabetic retinopathy. Invest Ophthalmol Vis Sci 1996;37:886–97. 8603873

[6] Guan K Hudson C Wong T Retinal hemodynamics in early diabetic macular edema. Diabetes 2006;55:813–8. 16505248 10.2337/diabetes.55.03.06.db05-0937

[7] Gilmore ED Hudson C Nrusimhadevara RK Retinal arteriolar diameter, blood velocity, and blood flow response to an isocapnic hyperoxic provocation in early sight-threatening diabetic retinopathy. Invest Ophthalmol Vis Sci 2007;48:1744–50. 17389507 10.1167/iovs.06-1016

[8] Hardarson SH Stefansson E Retinal oxygen saturation is altered in diabetic retinopathy. Br J Ophthalmol 2012;96:560–3. 22080478 10.1136/bjophthalmol-2011-300640

[9] Clermont AC Aiello LP Mori F Vascular Endothelial growth factor and severity of nonproliferative diabetic retinopathy mediate retinal hemodynamics in vivo: a potential role for vascular endothelial growth factor in the progression of nonproliferative diabetic retinopathy. Am J Ophthalmol 1997;124:433–46. 9323935 10.1016/s0002-9394(14)70860-8

[10] Miller JW Le Couter J Strauss EC Vascular endothelial growth factor a in intraocular vascular disease. Ophthalmology 2013;120:106–14. 23031671 10.1016/j.ophtha.2012.07.038

[11] Kroll P Rodrigues EB Hoerle S Pathogenesis and classification of proliferative diabetic vitreoretinopathy. Ophthalmologica 2007;221:78–94. 17380062 10.1159/000098253

[12] Zhang X Bao S Hambly BD Vascular endothelial growth factor-A: a multifunctional molecular player in diabetic retinopathy. Int J Biochem Cell Biol 2009;41:2368–71. 19646547 10.1016/j.biocel.2009.07.011

[13] Jorgensen CM Hardarson SH Bek T The oxygen saturation in retinal vessels from diabetic patients depends on the severity and type of vision-threatening retinopathy. Acta Ophthalmol 2014;92:34–9. 24330421 10.1111/aos.12283

[14] Jorgensen C Bek T Increasing oxygen saturation in larger retinal vessels after photocoagulation for diabetic retinopathy. Invest Ophthalmol Vis Sci 2014;55:5365–9. 25097242 10.1167/iovs.14-14811

[15] Hammer M Vilser W Riemer T Diabetic patients with retinopathy show increased retinal venous oxygen saturation. Graefes Arch Clin Exp Ophthalmol 2009;247:1025–30. 19404666 10.1007/s00417-009-1078-6

[16] Hardarson SH Retinal oximetry. Acta Ophthalmol 2013;91:1–47. 10.1111/aos.12086 23470088

[17] Rilven S Torp TL Grauslund J Retinal oximetry in patients with ischaemic retinal diseases. Acta Ophthalmol 2017;95:119–27. 27585711 10.1111/aos.13229

[18] Tayyari F Khuu LA Sivak JM Retinal blood oxygen saturation and aqueous humour biomarkers in early diabetic retinopathy. Acta Ophthalmol 2019;97:e673–9. 30690929 10.1111/aos.14016

[19] Moher D Shamseer L Clarke M Preferred reporting items for systematic review and meta-analysis protocols (PRISMA-P) 2015 statement. Syst Rev 2015;4:1. 25554246 10.1186/2046-4053-4-1 PMC4320440

[20] Rostom A Dube C Cranney A Celiac Disease. Rockville (MD): Agency for Healthcare Research and Quality (US); 2004 Sep. (Evidence Reports/Technology Assessments, No. 104.) Appendix D. Quality Assessment Forms.http://www.ncbi.nlm.nih.gov/books/NBK35156.

[21] Wells GA Shea B O’Connell D The Newcastle-Ottawa Scale (NOS) for assessing the quality if nonrandomized studies in metaanalyses [EB/OL] [2012-06-15]. Available at: http://www.ohri.ca/programs/clinical_epidemiology/oxford.htm.

